# Embryonic Porcine Skin Precursors Can Successfully Develop into Integrated Skin without Teratoma Formation Posttransplantation in Nude Mouse Model

**DOI:** 10.1371/journal.pone.0008717

**Published:** 2010-01-18

**Authors:** Zhenggen Huang, Junjie Yang, Gaoxing Luo, Chengjun Gan, Wenguang Cheng, Shunzong Yuan, Xu Peng, Jianglin Tan, Xiaojuan Wang, Jie Hu, Shiwei Yang, Yair Reisner, Liangpeng Ge, Hong Wei, Ping Cheng, Jun Wu

**Affiliations:** 1 State Key Laboratory of Trauma, Burn and Combined Injury, Institute of Burn Research, Southwest Hospital, Third Military Medical University, Chongqing, China; 2 Chongqing Key Laboratory for Proteomics of Diseases, Chongqing, China; 3 Department of Immunology, Weizmann Institute of Science, Rehovot, Israel; 4 Department of Zoology, Third Military Medical University, Chongqing, China; 5 Department of Clinical Laboratory Science, Third Military Medical University, Chongqing, China; Tufts University, United States of America

## Abstract

How to improve the wound healing quality of severe burn patients is still a challenge due to lack of skin appendages and rete ridges, no matter how much progress has been made in the fields of either stem cell or tissue engineering. We thus systematically studied the growth potential and differentiation capacity of porcine embryonic skin precursors. Implantation of embryonic skin precursors (PESPs) of different gestational ages in nude mice can generate the integrity skin, including epidermis, dermis and skin appendages, such as sweat gland, hair follicle, sebaceous gland, etc.. PESPs of embryonic day 42 possess the maximal growth potential, while, the safe window time of PESPs transplantation for prevention of teratoma risk is E56 or later. In conclusion, PESPs can form the 3 dimensional structures of skin with all necessary skin appendages. Our data strongly indicate that porcine embryonic skin precursors harvested from E56 of minipig may provide new hope for high-quality healing of extensive burns and traumas.

## Introduction

Many efforts have been made to reconstruct the physiological structures of skin after severe trauma or burn injury, but the result is far from the desired one, in spite of great progress made in the fields of both stem cells and tissue engineering. Embryonic stem cells can differentiate into all type of cells[1], but the precise control of differentiation is still a challenge[2]. Induced pluoripotent stem cells (iPS) are currently thought to be a promising alternative way for replacement of embryonic stem cells [3], [4], however, the risk of tumorigenesis and difficulty of forming the 3 dimentional structures of skin or organs encountered upon use of both approaches have yet to be overcome. On the other hand, tissue engineering is another promising way to repair the skin's defect. Unfortunately, current skin tissue engineering can only form “epidermis” and “dermis” without rete ridges, sweat gland, hair follicle, sebaceous gland, etc [5], which leads to poor mechanical strength and little radiating heating [6]. Recent reports have shown that at a certain gestational stage the organ-committed tissue precursors could safely generate kidney, pancreas, liver, spleen and lung without the risk of teratoma formation [7], [8], [9], [10]. These data has led us to test whether the skin precursors, if there are any, could also differentiate into integrity skin and exhibit a significant growth potential. We therefore assessed the growth potential of minipig embryonic skin precursors, the capacity of generating integrity skin and the possible safe window time for prevention of teratoma risk after transplantion of minipig embryonic skin precursors.

## Materials and Methods

### Animals

BABL/c nude mice about 4 to 6 weeks old were purchased from Vital River Lab Animal Technology Co., Ltd., and maintained under specific-pathogen-free conditions in Experimental Animal Department of the Third Military Medical University (Chongqing, China). Guizhou mini pigs (black skin, black hairs) were from closed colony, and maintained in Experimental Mini Pig Base of The Third Military Medical University (Chongqing, China). The protocols were approved by the Institutional Animal Care and Use Committee in Third Military Medical University.

### Preparation of Porcine Embryonic Skin Precursors (PESPs)

PESPs were obtained from pregnant Guizhou mini sows at precise stages of pregnancy (E35, E42, E56, E70 and E91). Fetal pigs were washed in cold normal saline (4–6°C). Warm ischemia time was less than 10 minutes. PESPs were harvested from the middle back of porcine embryo, immersed in cold (4–6°C) PBS (0.01 mol/L, pH 7.2–7.4), and then minced to microgranules at the size of 1–2 mm^2^ for implantation.

### Transplantation Model

Transplantation was performed in specific-pathogen-free operating room. BABL/c nude mice as the host were under general anesthesia with 1% pentobarbital at a dose of 5–8 ul/g via intraperitoneal injection. Each group had 12 mice that received the porcine embryonic skin precursors from a same gestational day. The designed groups were showed in Table 1. The dorsal part of the mouse was disinfected with iodophors. A size of 1.0 cm×2.0 cm full-thickness skin defect was made on the back through a median incision. Total 0.1 ml suspension of PESPs in PBS (20% of PESPs in PBS, v/v) was implanted onto the wound protected by adult white Bama mini pig skin pieces (purchased from Zongshen Junhui Biotech co.,Ltd). Penicilin and streptomycin (500 u each in 0.25 ml of normal saline) were intraperitoneally injected postimplantation.

**Table 1:** The appearance time of black color and black hair after PESPs posttransplantation.

Embryonic day	Time of appearance (week of posttransplantation)	
	Black color	Black hair
E35	4	7
E42	3	5
E56	2	4
E70	1–2	3
E91	1–2	2–3

### Observation of Graft Growth

The donor pig is black and haired, while the recipient mouse is white and hairless. Thus, we macroscopically observed the skin color and hair growth in the implanted area every day after transplantation for evaluation of the origin of regenerative tissues. Mice were sacrificed 12 weeks after transplantation, and long (L) and short (W) axes of the grafts were measured. The posttransplant size was calculated by multiplying L×W.

### Observation of Graft Differentiation

Teratoma was defined when tissue representatives of at least two germ layers were detected in tumor, growing within the implants[7]. Recipient mice were sacrificed at 6 and 12 weeks after transplantation. Graft samples were fixed with 4% formaldehyde, imbedded in paraffin, sectioned at the thickness of 5 µm and stained by hematoxylin/eosin (H&E) for histological observation.

### Determination of the Origin of Neoregenerative Tissues

Fontana stain was used to determine whether the tissue originated from Guizhou minipig that is the black one. Briefly, tissues were fixed in 4% formaldehyde and embedded in paraffin. Paraffin sections were deparaffinized, and were rinsed in Fontana silver solution at room temperature away from light for 18–48 hours, and placed in 0.2% gold chloride solution, in 5% Na_2_S_2_O_3_ solution, and counterstained by Van Gieson solution, followed by dehydrated, transparented, and mounted steps. Melanin and argyrophilic granules should be black, keratin orange, and others red.

Immunohistochemistry. As anti-Cytokeratin MNF 116 (DAKO, Code: M0821) is specific for pig epithelium, and is nonreactive with mouse epithelia, we used it for outlining pig fetal skin epithelium. Moreover, anti-Vimentin V9 (DAKO, Code: N1521) is specific for pig mesenchyma, and is non reactive with mouse mesenchymal cells, we thus used it for detecting fibroblasts, adipocytes, muscle cells, as well as blood vessel endothelium of pig origin. Briefly, paraffin sections were deparaffinized, washed in PBS 3 time, followed by incubation with 3% H2O2 for 15 minutes and PBS washing again. Then the sections were incubated in 10% normal goat serum (Zhongshan Biology Company, Beijing, China) for 45 minutes, followed by incubation with either anti-Cytokeratin MNF116 antibody at the dilution of 1∶200, or anti-Vimentin V9 at the dilution of 1∶10) overnight at 4°C. washed 4 times in PBS containing 0.1% Tween-20 (PBST) for 3 min. All tissue sections were incubated with biotinylated goat-anti-mouse IgG antibody at the dilution of 1∶200 for 1 hour at room temperature, followed by avidin peroxidase reagent (Zhongshan Biology Company, Beijing, China). Diaminobenzidine was used as the chromogen. Tissue sections were counterstained with Hematoxylin.

### Statistics

All data were presented as mean ± SD, and evaluated by one-way ANOVA. P value less than 0.05 was considered as statistic significance.

## Results

### Organ Specific Development of PESPs

#### The physical characteristics and histology of PESPs at different embryonic stages

**Figure 1:**
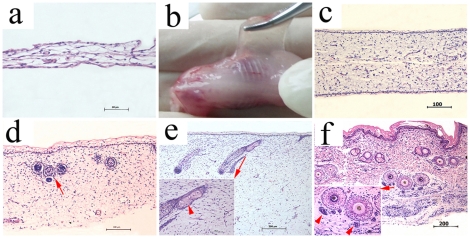
Developmental histology of E35, E42, E56, E72 and E91. (a) The histological image of E35. (b) The naked image of E42. (c) The histological image of E42. (d) The histological image of E56. Hair follicle-like structure is indicated by arrow. (e) The histological image of E72. The sebaceous gland is indicated by arrow head. (f) The histological image of E91. The duct of sweat gland is indicated by arrow head.

PESPs from E35 were tremelloid, transparent and fragile. It was impossible to distinguish the dermal and the epidermal sides of the removed samples by naked eyes. H&E stain showed that PESPs from E35 were covered by a single layer of cells. The inferior connective tissue was extremely loose, and no vessel or other skin appendages could be observed (Figure 1a). The PESPs from E42 was tremelloid, semitransparent and fragile (Figure 1b). The PESPs from E42 was covered by 2–3 continuous layers of cells. Microangiums were observed in dermis without visible hair follicle or dermal papilla (Figure 1c). PESPs from E56 had the appearance of frosted glass. The number of out layers increased noticeably up to 10 layers. Hair follicles-like structure and dermal papillas could be found. Small vessels could be seen in dermis as well (Figure 1d), but Fortana stain was negative. Black color could be observed by naked eyes in PESPs from E70, which was also Fortana stain positive. At this stage, sebaceous glands were found, and vessels were apparent in dermis. However, no sweat gland or hair was observed (Figure 1e, sebaceous gland was indicated by arrowhead in the magnified image). PESPs from E91 were already black and owned visible hairs (Figure 1f). There were obvious rete ridges, sebaceous glands, sweat glands (sweat gland ducts were indicated by arrowhead in the magnified image) and keratinization at the out layer.

#### Macroscopic observation of the growing embryonic skin implants

**Figure 2:**
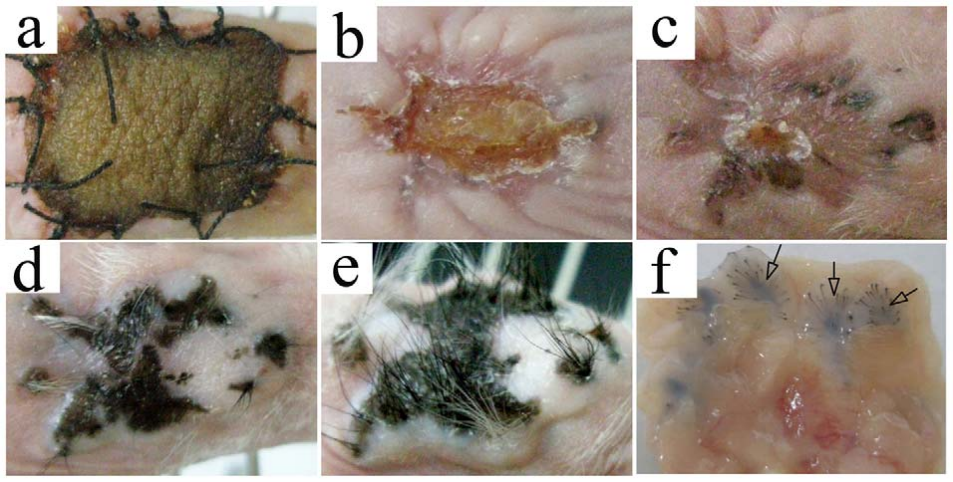
The growth of E56-old PESPs after implantation. (a) The PESPs were grafted on the wound and protected by the adult white BAMA pini-pig skin. (b and c) The wound was healed first by host mouse epidermis 2 and 3 weeks postimplantation. (d and e) After 4 weeks of implantation, black color became visible. Finally, the black hair grew out. (f) The dermal side of the black after 6-week implantation.

In the model of implantation of PESPs, the wound was healed first by mouse epidermal cells (Figures 2b and 2c). However, 2 to 3 weeks posttransplantation, some black spots and nodules could be observed and touched, respectively, under the healed wound. Black spots and hairs could be observed in all PESPs in 4–6 weeks after transplantation (Figures 2d and 2e). One granule of skin precursor could form a skin island like a sucker (Figure 2f). During 8th–12th week posttransplantation, the skin islands had been fusing together. From 8 weeks posttransplantation, the black hairs could be easily found in the wound area (Figure 2e). The growth speed of all implanted skin precursors obviously slowed down after 12 weeks posttransplantation. There was no significant difference in gross morphology of posttransplants between any two gestational age groups, except the time of appearance of the black color and the black hairs in the wound area (Table 1).

#### Histology of the growing embryonic skin implants

**Figure 3:**
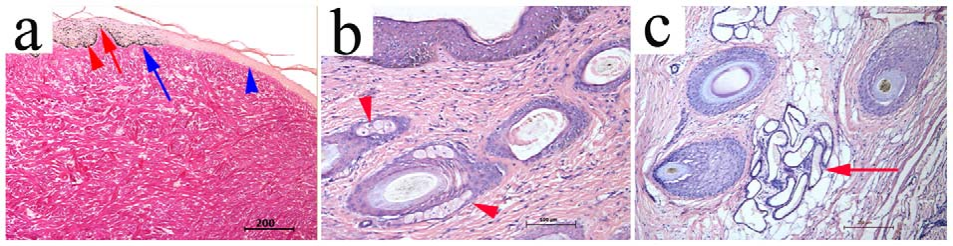
The histology of E56-old PESPs after 12-week implantation. (a) The neoregenerative tissue containing melanin was stained by means of Fontana (indicated by arrow). The epidermal and dermal rete ridges were shown by arrowhead. (b) The sebaceous gland was indicated by arrowhead. (c) The sweat gland ducts were indicated by arrow.

Histological examination showed that the cambiums from E56 implants contained epidermis and dermis, including apparent dermal papillas and rete redges (Figure 3a). Importantly, there were apparent hair follicles (Figure 3b and 3c), sebaceous glands (Figure 3b) and sweat gland ducts (Figure 3c) in dermis.

### The Origin of Neoregenerative Tissues

**Figure 4:**
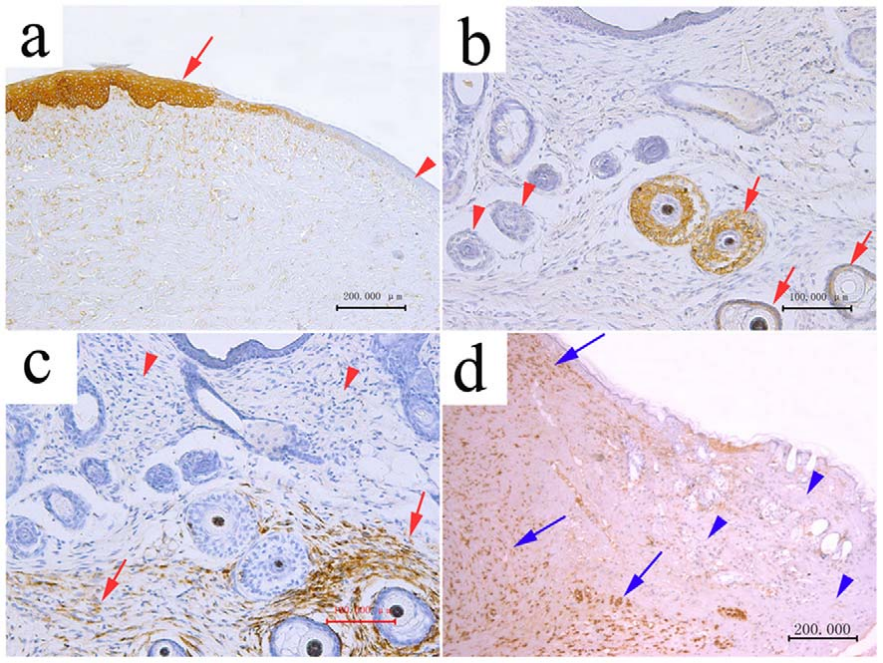
The immunohistological staining of neoregenerative tissues after 6–12 week implantation of E56 PESPs. (a) Cytokeratin MNF116 positive cells were located in epithenium (12-week postimplantation). (b) Cytokeratin MNF116 positive cells were located in hair follicle sheet (6-week postimplantation). (c) Vimentin 9 positive cells were located in the dermis around hair follicles (6-week postimplantation). (d) Vimentin 9 positive cells were located in the deep dermis tissue under the host tissue (12-week postimplantation).

Fontana stain showed that there were melanin cells in the neoregenerative epidermis (Figure 3a). Furthermore, the neoregenerative epithenium and hair follicle sheet cells were positive for anti-cytokeratin MNF116 (Figures 4a and 4b). Dermal cells around hair follicles and in the deep dermal tissue were positive for anti-vementin 9 (Figures 4c and 4d). Neo-regenerative tissues were completely fused with host mouse tissues.

### Teratoma Development

**Figure 5:**
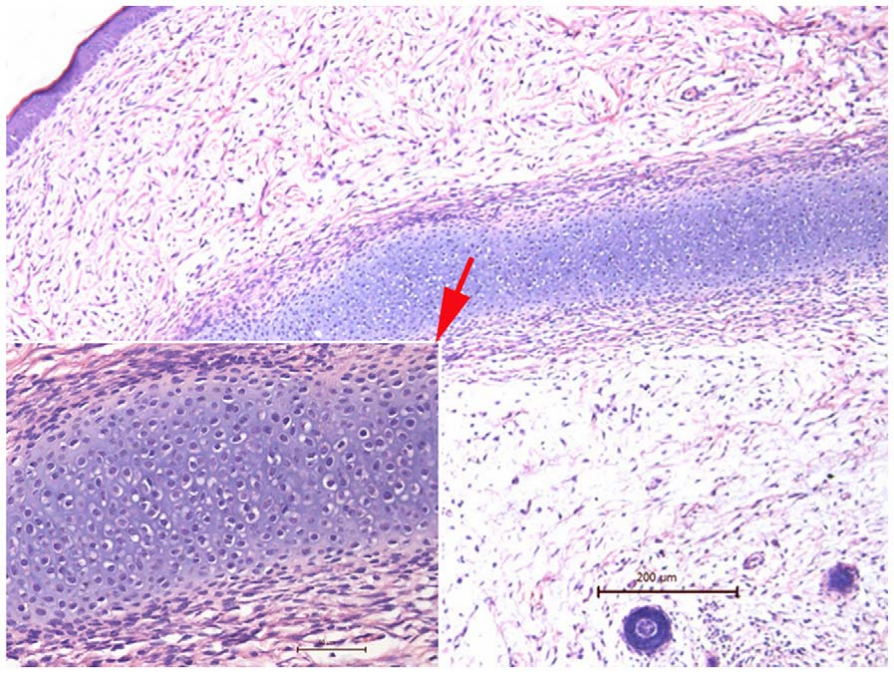
The teratoma-like structure of E42-old PESPs after 6-week implantation. Cartilage tissue and cutaneous tissue were found in neoregenerative tissues (HE, ×200 for larger photo, ×400 for smaller photo).

The possibility of PESPs differentiation into teratoma was tested in our experiments. The histological examination of implanted skin precursors after 12 weeks of implantation showed that PESPs obtained from E42 could differentiate into both cutaneous tissues and cartilage tissue (2 out of 12 mice, 3 out of 50 grafts) (Figure 5). No teratoma was found following implantation of in E56 or later skin precursor grafts.

### The Growth Potential of PESPs

**Figure 6:**
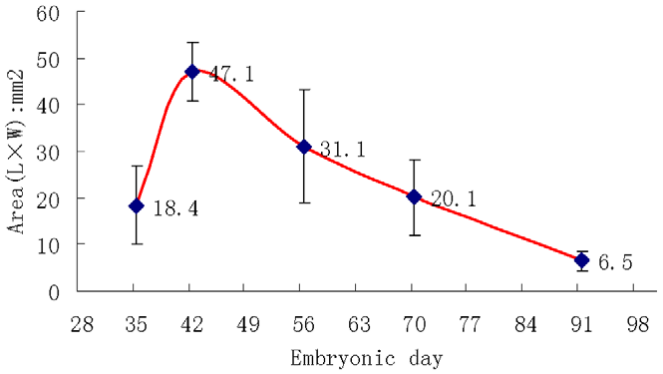
Growth potential of PESPs from different gestational ages posttransplantation. E42-old PESPs possessed the maximal growth potential. There were significant difference between E42 and E35 (p = 0.001), E42 and E70 (p = 0.001), E42 and E91 (p = 0.001). The number of animals at each time point was 12. There was no significant difference between E42 and E56 (p = 0.21).

Generally, the growth potential of PESPs increases firstly and then decreases along with its gestational age. PESPs from E42 showed the maximal growth potential (47.1±6.3 mm^2^) that was significantly higher than that exhibited by tissues harvested at other gestational ages (Figure 6).

## Discussion

The challenge of repairing a skin defect entails restoration of the cutaneous anatomical structures and functions. The current strategies to achieve the above aims are based on epidermal stem cell implantation, engineered skin covering and microskin grafting. Epidermal committed stem cells have been successfully isolated and cultured to form in vitro the epidermal sheet that can be grafted for covering the wound[11]. However, the cultured epidermal sheet without dermis is very fragile, and the final outcome of grafting cultured epidermal sheet on burn wounds is not satisfactory, particularly on deep burn injury wounds[6]. Embryonic stem cells, which exhibit a universal differentiation capacity, could theoretically differentiate into any mature tissue cells[1], [2], [12]. But so far, the precise differentiation, the efficiency of differentiation and the risk of tumorigenesis hamper the application of embryonic stem cells in tissue rehabilitation. Hair follicle stem cells located in external root sheaths were defined as stem cells of skin, with the ability to differentiate into hair follicles, sebaceous glands and keratinocytes. Tissue engineered skin using hair follicle stem cells can reconctruct the bilayer structure of skin, but cannot reconstruct skin appendages effectively[5]. Moreover, like other embryonic stem cells, the resource of hair follicle stem cells is very limited and has to proliferate in order to attain a curative potential. Therefore, the current engineered skin can only partly reconstruct the skin functions that still lack skin appendages. Clinically, microskin transplantation is still an effective method to treat extensive burns and traumas, but due to lack of dermis, especially the papillar layer, hair follicles, sweat glands and other skin appendages, the neoregenerative skin possesses poor mechanical strength and elasticity, and cannot sweat. Thus, an effective reconstruction of the structure and function of the skin remains a major challenge.

Interestingly, a recent series of studies demonstrated that grafting embryonic tissue or organ precursors could successfully generate the wanted tissues or organs without the risk of teratoma formation[7], [8], [9], [13], [14], [15], [16]. This strategy overcomes both the difficulties of precise control of stem cell differentiation and the formation of 3 dimensional structures of a tissue or an organ. These precursors compromise all the information of organogenesis. Thus, the working hypothesis of the present study was that there could be also a stage during embryonic development at which skin precursors could generate the integrity skin consisting of epidermis, dermis and all necessary skin appendages and rete ridges. Our data clearly demonstrate for the first time that all the PESPs of E35-E91 exhibit the capacity to grow and generate the integrity skin with epidermis, dermis, rete ridges and appendages, such as hair follicles, sweat glands and sebaceous glands. However, different growth potential and differentiation capacity are exhibited by tissues harvested at different gestational time points. The results show that maximal growth potential is found upon implantation E42 old skin precursor tissue (initial size *vs* final size: 1 to 2 mm^2^ *vs* 47 mm^2^) while tissue harvested at later time points are gradually lose their capacity to grow (Figure 5). The surprising finding that the maximal growth potential is not found at the earliest PESPs, indicates that the wound environment favors the growth of more mature skin precursors, although the earlier skin precursors might possess more growth potential in situ. Another important issue addressed by our study is the risk of teratoma formation after PESPs transplantation. Previous studies by Reisner et al. demonstrate that the safe window time of various organ precursors for transplantation is different[7], [8], [9]. Herein we show that the optimal gestational age for skin precursor implantation is E56, which is similar to lung precursors. The E56 skin precursors compromise all the skin-genesis information that specifically, directly instructs the skin precursors to generate the integrity skin without the risk of teratoma formation.

Clearly, considering that our study was performed in immunodeficient recipients, further studies in immune competent animals are required before PESPs transplantation could be applied to humans. Previous studies in rodents indicate that local immune tolerance might be induced if small pieces of human allo- and auto-skin tissues were grafted in an intermingled way[17], or if skin grafts were used from genetically modified transgenic pig donors over-expressing the CTLA4Ig gene[18], [19]. Further experiments using these approaches in a preclinical nonhuman primate model are warranted.

In conclusion, the present study demonstrates that porcine embryonic skin precursors can generate the integrity of skin that includes epidermis, dermis, rete ridges and skin appendages. This work also defines the optimal gestational age for skin precursor implantation. Our data provide the feasibility for physiological reconstruction of skin defects, which is important not only to the treatment of burns but also in the field of cosmetics.
